# Supplementary material for: Learning curve and surgical outcome of robotic assisted colorectal surgery with ERAS program
Source: Sci Rep. 2022 Nov 29;12:20566. doi: 10.1038/s41598-022-24665-w (PMC9709162; doi:10.1038/s41598-022-24665-w)
Supplement: Supplementary file 2 — Supplementary Information 2. [file 41598_2022_24665_MOESM2_ESM.zip › RAL Raw data-Ñ[▒K/117/OP N_ñw╝╨░O▒Kñσ.pdf]

請選擇...reporter

# 臺中榮民總醫院
## 手術室護理記錄

[redacted]

### 基本資料

- 室別： 開刀房-17室，手術科別： CRS，手術類別： 常規，日期： 2021/10/09
- 進入時間： 0755 ； 麻醉開始時間： 0805 ； Time Out時間： 0950 ； 劃刀時間： 0951 ； 手術結束時間： 1330 送出時間： 1415 ；
- 麻醉方式： GE
  診斷：S COLON CA FOR OP
  手術名稱：Robot Assisted Colectomy(L)
  手術局部用藥：nil
  術前預防性抗生素：
  - 抗生素名稱一： Veterin 1GM IVD由麻醉護理人員給予
  - 時間： 0920
  ，追加預防性抗生素：
- 手術醫師： 蔣鋒帆，陳瑋夫，葉達昇
- 麻醉醫師： VS 張詒婷
- 
  - 刷手護士A ： 羅媗鈴，時間： 0755
  - 流動護士A ： 劉懿潔，時間： 0755，接替流動一： 陳慧瑜，時間： 1100，接替流動二： 劉懿潔，時間： 1130

---

### 皮膚狀況

A-V Shunt： 無

術前： 不完整

- 7. 發紅

部位(術前)：

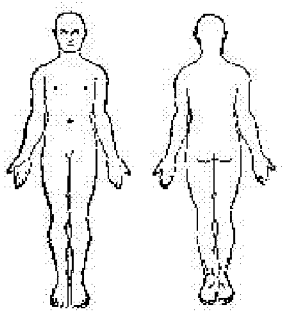

右頸側及右手臂各一處發紅

記錄者： 上次簽章: 劉懿潔

術後： 不完整

部位(術後)：

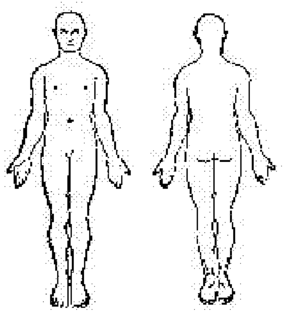

如術前

記錄者：

## 手術準備

手術臥式 單一手術臥式

- 
  - 使用附件： 溫毯 38 ℃，頭圈，腳架
  - 皮膚準備： 10%Aq Betadine，Hibiscrub
  - 電燒： 使用電燒: 電極片位置： 其它： 迴路電極墊，記錄者： 上次簽章: 劉懿潔

止血帶T： 無

## 植入物

無

## 標本送檢

- 細菌培養 無
- 細胞學 無
- 冰凍切片 無
- 病理標本
  組織名稱一：S-Colon
  ,
  組織名稱二：Distal cut end
  ,
  組織名稱三：IMA root
- 截肢 無

標本交付 標本： 3 袋，標本： 0 盒，標本： 0 瓶，細胞學： 0 管，Culture Tube： 0 管，被交付醫師： CR 陳璿夫，

- 記錄者： 上次簽章: 劉懿潔
- 時間： 1300

## 傷口術後敷料

傷口敷料：　　　　傷口敷料： 無

- 請於圖上標示部位：

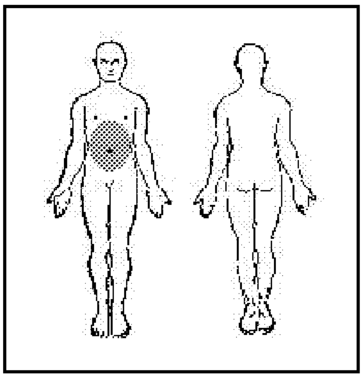

- 傷口敷料：mepore

## 導管

| 導管 | 天數 | 導管資訊 | 插入日期 | 插入時間 | 更換日期 | 拔除日期 | 拔除時間 | 拔除原因 | 預計拔除/更換 | 預計拔除/更換日期 |
|---|---|---|---|---|---|---|---|---|---|---|
| A-line (1) | 1 | 號碼:20 G; 部位:左手; | 2021/10/09 | 0820 | | | | | | |
| Foley(1) | 1 | 號碼:16 Fr.; 部位:膀胱; | 2021/10/09 | 0830 | | | | | | |

## 用物計數

- 手術中出血量： 少 c.c.
- 手術中尿量： 280 c.c.
- 
  - 其它： 無

用物計數： 單一切口

- 1.器械： 正確，2.縫針： 正確，3.刀片： 正確，4.紗布： 正確，6.紗球： 未使用，7.PEANUTS ： 未使用，8.紗條： 未使用，9.絲帶： 未使用，10.針頭： 未使用，11.Vessel loop： 未使用，12.Boots： 未使用，13.Jelco： 未使用，14.棉條： 未使用，
    - 第一次：
        - 刷手護士 ： 羅媗鈴
        - 流動護士： 上次簽章: 劉懿潔
        - 時間： 1225

      ,
    - 第二次：
        - 刷手護士 ： 陳慧瑜
        - 流動護士： 上次簽章: 劉懿潔
        - 時間： 1240

      ,
    - 第三次：
        - 刷手護士 ： 羅媗鈴
        - 流動護士： 上次簽章: 劉懿潔
        - 時間： 1300

## 備註

備註：NIL

- 記錄者： 上次簽章: 劉懿潔
- 時間： 0810

填寫護理人員 劉懿潔

正式摘要

請選擇...reporter

doctorRank:B
